# Axonal Segregation and Role of the Vesicular Glutamate Transporter VGLUT3 in Serotonin Neurons

**DOI:** 10.3389/fnana.2016.00039

**Published:** 2016-04-12

**Authors:** Aurore N. Voisin, Ouissame Mnie-Filali, Nicolas Giguère, Guillaume M. Fortin, Erika Vigneault, Salah El Mestikawy, Laurent Descarries, Louis-Éric Trudeau

**Affiliations:** ^1^Department of Pharmacology, Faculty of Medicine, GRSNC, Université de MontréalMontreal, QC, Canada; ^2^ Department of Neurosciences, Faculty of Medicine, GRSNC, Université de MontréalMontreal, QC, Canada; ^3^Department of Psychiatry, Douglas Hospital Research Center, McGill UniversityMontreal, QC, Canada; ^4^Institut National de la Santé et de la Recherche Médicale (INSERM), UMR-S 1130Paris, France; ^5^Centre National de la Recherche Scientifique (CNRS), UMR 8246Paris, France; ^6^Institut de Biologie Paris-Seine (IBPS), Sorbonne Universités, Université Pierre et Marie Curie (UPMC) Paris, UM119 Neuroscience Paris SeineParis, France

**Keywords:** vesicular glutamate transporter, cotransmission, serotonin, neuronal survival, serotonin transporter

## Abstract

A subset of monoamine neurons releases glutamate as a cotransmitter due to presence of the vesicular glutamate transporters VGLUT2 or VGLUT3. In addition to mediating vesicular loading of glutamate, it has been proposed that VGLUT3 enhances serotonin (5-HT) vesicular loading by the vesicular monoamine transporter (VMAT2) in 5-HT neurons. In dopamine (DA) neurons, glutamate appears to be released from specialized subsets of terminals and it may play a developmental role, promoting neuronal growth and survival. The hypothesis of a similar developmental role and axonal localization of glutamate co-release in 5-HT neurons has not been directly examined. Using postnatal mouse raphe neurons in culture, we first observed that in contrast to 5-HT itself, other phenotypic markers of 5-HT axon terminals such as the 5-HT reuptake transporter (SERT) show a more restricted localization in the axonal arborization. Interestingly, only a subset of SERT- and 5-HT-positive axonal varicosities expressed VGLUT3, with SERT and VGLUT3 being mostly segregated. Using VGLUT3 knockout mice, we found that deletion of this transporter leads to reduced survival of 5-HT neurons *in vitro* and also decreased the density of 5-HT-immunoreactivity in terminals in the dorsal striatum and dorsal part of the hippocampus in the intact brain. Our results demonstrate that raphe 5-HT neurons express SERT and VGLUT3 mainly in segregated axon terminals and that VGLUT3 regulates the vulnerability of these neurons and the neurochemical identity of their axonal domain, offering new perspectives on the functional connectivity of a cell population involved in anxiety disorders and depression.

## Introduction

Mounting evidence has accumulated in recent years demonstrating that a subset of monoamine neurons including 5-HT, dopamine (DA) and epinephrine (E) and norepinephrine (NE) neurons uses glutamate as a second neurotransmitter (Mestikawy et al., 2011; Hnasko and Edwards, 2012; Trudeau et al., 2014; Zhang et al., 2015). In DA, E and NE neurons, glutamate packaging is mediated by the type 2 vesicular glutamate transporter (VGLUT2; Stornetta et al., 2002; Dal Bo et al., 2004), while in serotonin (5-HT) neurons, this vesicular uptake is mediated by VGLUT3 (Gras et al., 2002). Although in each of these neuronal classes glutamate has been demonstrated to act as a fast synaptic neurotransmitter (Sulzer et al., 1998; Varga et al., 2009; Stuber et al., 2010; Tecuapetla et al., 2010; Holloway et al., 2013), other roles of glutamate release by these neurons have also been proposed. For example, VGLUT2-mediated glutamate release has been proposed to play a critical developmental role in DA neurons, contributing to axonal growth and to neuronal survival (Fortin et al., 2012). VGLUT3 has also been suggested to enhance the vesicular packaging of 5-HT in raphe neuron axon terminals through a functional interaction between the vesicular monoamine transporter (VMAT2) and VGLUT3 (Amilhon et al., 2010), a mechanism of vesicular synergy first demonstrated between VGLUT3 and the vesicular acetylcholine transporter (VAChT; Gras et al., 2008). Whether such additional roles of the glutamatergic co-phenotype are conserved across DA, 5-HT and NE neurons is unclear. For example, it has not been directly determined whether VGLUT3 contributes to development and survival of 5-HT neurons.

It has been observed that a large proportion of central 5-HT neurons expresses VGLUT3 (Fremeau et al., 2002; Gras et al., 2002; Schäfer et al., 2002; Takamori et al., 2002; Hioki et al., 2010; Calizo et al., 2011; Kiyasova et al., 2013). These neurons are most abundant in the dorsal and medial raphe nuclei (DRN and MRN, respectively) and provide dense 5-HT innervation throughout the forebrain (particularly to the striatum, lateral septum, dorsal and ventral hippocampus) and brainstem (Dahlström and Fuxe, 1964). The distribution of VGLUT3 in the axonal arborization of 5-HT neurons is unclear. However, previous data argue for a heterogeneous distribution in a subset of axon terminals established by 5-HT neurons. For example, in the DRN, VGLUT3 has been reported to be present in only a subset of axonal collaterals (Commons, 2009). Similarly, in forebrain nuclei, only a variable proportion of 5-HT axonal fibers are reported to be VGLUT3-immunoreactive (Shutoh et al., 2008). It has also been demonstrated that 5-HT axonal terminals expressing VGLUT3 are only rarely co-labeled for the 5-HT reuptake transporter (SERT), a protein often found in serotonergic axon terminals (Gras et al., 2002; Amilhon et al., 2010). Due to the presence in most of these regions of VGLUT3-immunoreactive axonal projections originating from non-5-HT neurons, it remains unclear whether the axonal arborization of individual 5-HT neurons contains domains where VGLUT3 is segregated from typical 5-HT neuron markers such as SERT, tryptophan hydroxylase (TrypH) or 5-HT itself. Interestingly, qualitative evidence has been previously provided suggesting the possibility of a partial segregation of VGLUT3 and TrypH along the axonal domain of embryonic rat raphe neurons in culture (Schäfer et al., 2002).

In the present work, we aimed to address these outstanding critical issues. We first used a postnatal mouse raphe neuron culture model to examine the axonal arborization of individual 5-HT neurons to determine whether VGLUT3 is found at release sites that are distinct from those containing 5-HT or SERT. We also used this model to test the hypothesis that, as suggested for VGLUT2 in DA neurons, VGLUT3 and glutamate release play a developmental role in 5-HT neurons. We found that *in vitro*, the expression of SERT and VGLUT3 was largely segregated in different axonal domains of individual 5-HT neurons, a conclusion supported by the finding that in *vivo*, these proteins are rarely colocalized. Using a VGLUT3 knockout (KO) mouse line, we also found that deletion of VGLUT3 increased the vulnerability of 5-HT neurons in culture and reduced the density of 5-HT-immunoreactivity in terminals of both the dorsal striatum and dorsal hippocampus.

## Materials and Methods

### Animals

All procedures involving animals and their care were conducted in accordance with the *Guide to Care and Use of Experimental Animals* of the Canadian Council on Animal Care. The experimental protocols were approved by the animal ethics committees of the Université de Montréal.

All experiments were performed using wild type (WT) or homozygous VGLUT3 KO mice obtained by crossing *Vglut3*^−/+^ mice (Gras et al., 2008). Mice were housed together in groups of 2–4 per cage in a temperature-controlled room with a 12:12 h dark/light cycle. Food and water were provided *ad libitum*. Age-matched WT littermates were used as controls for VGLUT3 KO mice. Only males were used. Animals were used between 45 and 75 days of age, except for the electron microscopy experiments, where 15 and 60–70 day old mice were compared.

### Tissue Processing and Cell Culture

Postnatal day 0–3 (P0–P3) mice were cryoanesthetized and decapitated for tissue collection. Primary cultures of raphe 5-HT neurons were prepared using a protocol identical to that used to prepare mesencephalic neurons, as previously described (Fasano et al., 2008). Raphe cells were plated onto monolayers of astrocytes. Cultures were used for immunocytochemistry at 1, 3 or 7 days *in vitro* (DIV).

### Immunocytochemistry on Cell Cultures

Cells were fixed with 4% paraformaldehyde (PFA), permeabilized, and nonspecific binding sites blocked. To identify 5-HT neurons and analyze their development, a rabbit anti-5-HT antibody was used at a dilution of 1:2000 (Immunostar). The same rabbit anti-5HT antibody was also used in combination with a monoclonal mouse anti-microtubule associated protein 2 (MAP2) antibody (1:2000, Sigma-Aldrich) to distinguish between the axons and dendrites of 5-HT neurons. To study the segregation of serotoninergic and glutamatergic terminals established by 5-HT neurons, the rabbit anti-5-HT antibody was used in combination with a goat anti-SERT antibody (1:1000; C-20, Santa Cruz Biotechnology) and a guinea-pig anti-VGLUT3 antibody (1:3000; Amilhon et al., 2010; Sakae et al., 2015).

### Immunohistochemistry

Male mice were deeply anesthetized with chloral hydrate (400 mg/kg, i.p.) and fixed by intracardiac perfusion using 150 ml of 4% PFA. The brain was removed, post-fixed by immersion for 24–48 h in PFA solution at 4°C, and washed in phosphate-buffered saline (PBS; 0.9% NaCl in 50 mm phosphate buffer, pH 7.4). Coronal 100 μm sections were cut using a VT1000S vibrating microtome (Leica Microsystems). Coronal sections were permeabilized, nonspecific binding sites blocked and incubated overnight with rabbit anti-5HT antibody (1:2000; Immunostar) in combination with goat anti-SERT antibody (1:1000; C-20, Santa Cruz Biotechnology) and guinea-pig anti-VGLUT3 antibody (1:3000; Amilhon et al., 2010; Sakae et al., 2015); these were subsequently detected using FITC-conjugated AffiniPure donkey anti-rabbit IgG, CY5-conjugated AffiniPure donkey anti-goat IgG and CY3-conjugated AffiniPure donkey anti-guinea-pig IgG (1:500; Jackson Immunoresearch).

### Image Acquisition with Confocal Microscopy

All of the *in vitro* and *in vivo* fluorescence imaging quantification analyses were performed on images captured using confocal microscopy. Images were acquired using an Olympus Fluoview FV1000 confocal microscope (Olympus). Images acquired using 488, 546 and 633 nm laser excitation were scanned sequentially to prevent nonspecific bleed-through signal. For the quantification of 5-HT neuron survival, 21 coverslips from four different cultures, fixed after 1 or 7 DIV, were acquired using a 20× water immersion objective. For the *in vitro* quantification of VGLUT3 expression in SERT neurons, five fields per coverslip with at least two cell bodies were randomly selected. Images were captured using a 20× water immersion objective from 12 coverslips coming from three different cultures at each time points (1, 3 and 7 DIV). For the *in vitro* quantification of 5-HT, SERT and VGLUT3 colocalization, 10 axonal fields per coverslip were randomly selected. Images were captured using a 60× oil-immersion objective from 12 coverslips coming from three different cultures at each time points (1, 3 and 7 DIV). For *in vivo* slice quantification, images were captured using a 60× oil-immersion objective from fields in the dorsal striatum, lateral septum, hippocampus or raphe. For image acquisition in the dorsal striatum and lateral septum, four random fields on each side were taken from the left and the right hemisphere in each section. For acquisition in the hippocampus, two fields were selected from the left and the right hemisphere in each section. For acquisition in the raphe, two fields were acquired from the dorsal and medial sub-regions in each section.

### Quantification of Confocal Images

All image quantification was performed using ImageJ (National Institutes of Health) software. A threshold was first applied at the same level for every image analyzed before performing further analyses. This threshold was determined by measuring the average background signal intensity and subtracted from the analyzed signal. The values from five different 5-HT neurons were averaged for each coverslip. At least 12 coverslips from three different cultures for each DIV were used for all quantifications. Quantifications of 5-HT- and VGLUT3-positive axon terminals in dorsal striatum and lateral septum sections were performed by averaging seven or eight images from seven different sections in each mouse. For hippocampal sections, the analysis was performed by averaging four images originating from six or seven different sections per animal. For raphe sections, the analysis was performed by averaging two images originating from six or seven different sections per animal.

### Double Fluorescence *In Situ* Hybridization (FISH)

Antisense cRNA riboprobes were obtained from the transcription of a PCR template amplified with primers containing the T7 promoter followed by the serotonin transporter (Slc6a4, SERT) sequence (forward primer 5′-AATTAACCCTCACTAAAGGGATGATGGTGACCAGTGTGGTGAACTGCAT-3′ and reverse primer 5′-TAATACGACTCACTATAGGGAGATCCATGAGAACAAACACGGG-3′) or the VGLUT3 sequence (forward primer 5′-AATTAACCCTCACTAAAGGGAGAAAAACAGGACTGGGCTGACCC-3′ and reverse primer 5′-TAATACGACTCACTATAGGGAGAGAGACCAAGGTCCATATTCCC-3′). SERT and VGLUT3 riboprobes were labeled with UTPs coupled to fluorescein and digoxigenin (DIG), respectively (Roche Applied Science). Brainstem coronal cryosections (10 μm) were fixed with 4% formaldehyde and hybridized as previously described (Gras et al., 2002). Sections were incubated with anti-fluorescein antiserum coupled with horseradish peroxidase (HRP; 1:250, 1 h at room temperature (RT), Roche Applied Science). The signal was amplified with the TSA-plus-biotin kit (Perkin Elmer). VGLUT3 RNA was visualized with Neutravidin Oregon Green (Invitrogen) at 488 nm excitation. After the HRP was inactivated with a glycine solution, the brain slices were incubated in the blocking solution for 30 min at RT. The slices were then incubated with anti-DIG coupled with HRP (1:2500, Roche Applied Science) for 1 h at RT. The TSA-plus-Cyanine 3 kit (Perkin Elmer, 10 min at RT) was used to detect the SERT transcript under 555 nm excitation fluorescence light. The slices were mounted with Fluoromount-G (Southern Biotech), scanned with a NanoZoomer 2.0-HT scanner (Hamamatsu Photonics), digitized with version 2.3.27 NDP.scan and finally visualized with NDP.view2. The number of SERT-positive neurons was estimated on serial sections taken in the dorsal and median raphe of VGLUT3 WT (*n* = 5) or KO (*n* = 4) mice.

### Stereological Analysis

Mice were perfused transcardially and brains were fixed in 4% PFA and cryoprotected in 10% sucrose before cryosectioning using a freezing microtome (Smith et al., 2003). Serial coronal free-floating sections (40 μm thickness) of the raphe were collected in an antifreeze solution. Sections were first rinsed in 0.01 M PBS, then in 0.01 M PBS + 30% H_2_O_2_ and again three times in 0.01 M PBS. Sections were then incubated in rabbit 5-HT antibody (Immunostar) at a 1:2000 dilution (all dilutions made in 0.3% Triton X-100/0.01 M PBS) for 48 h at 4°C. After rinses in 0.01 M PBS (3 × 10 min), they were incubated for 12 h at 4°C in biotin-streptavidin conjugated AffiniPure goat anti-rabbit IgG (1:200; Jackson Immunoresearch), washed three times with 0.01 M PBS and then incubated for 3 h at RT in streptavidin HRP conjugate (1:200; GE Healthcare). Sections were visualized after 10 min 3,3′-diaminobenzidine tetrahydrochloride (Sigma-Aldrich)/glucose oxidase reaction, mounted on charged microscope slides in 0.1 M acetate buffer, defatted using a series of ethanol and xylene baths and finally coverslipped using Permount.

The number of 5-HT neurons in the raphe was analyzed by unbiased stereological estimates. The total number of 5-HT neurons in the raphe was obtained by applying the optical fractionator method (Gundersen et al., 1988) using Stereo Investigator (version 6; MicroBrightField). In brief, the rostrocaudal extent of the raphe was examined in 5-HT-immunostained 40-μm thick coronal serial sections prepared from 2-months old WT and KO VGLUT3 mice, with the observer blind to the genotype. 5-HT-immunoreactive neurons were counted in every sixth section at 100× magnification using a 60 × 60 μm^2^ counting frame. The sections counted corresponded to atlas levels −4.36, −4.48, −4.60, −4.72, −4.84 and −4.96 mm (240 μm interval) with respect to bregma (Paxinos and Franklin, 2008). After immunohistochemistry, mounting, defatting and coverslipping, the mean section thickness, as measured with a *z*-axis microcator, was 12 μm. A 8 μm optical dissector was used with two 1 μm guard zones, and counting sites were located at 100 μm intervals after a random start.

### Quantitative Colocalization Analysis

Quantitative colocalization analysis of 5-HT, SERT and VGLUT3 proteins was performed by quantifying the Mander’s overlap coefficient (MOC) and overlap coefficients M1 and M2 (Manders et al., 1993). The MOC represents the degree of colocalization. Overlap coefficients M1 and M2 divide the value of colocalization into two separate parameters. They reflect the percentage of the total signal that colocalizes with each of the two immunoreactive signals. In the present work, coefficient M1 indicated a contribution of the first protein indicated in the figure caption, while coefficient M2 represented the contribution of the second protein.

#### Electron Microscopy

In order to obtain an optimal compromise between ultrastructural preservation and immunocytochemical detection, we used in the present study a procedure for preparation of mouse tissue for immunoelectron microscopy that was previously described in detail (Bérubé-Carrière et al., 2009). Briefly, mice were anesthetized with sodium pentobarbital (80 mg/kg i.p.) and then fixed by intracardiac perfusion of a solution of 3% acrolein in 0.1 M PBS (pH 7.4; 50 mL for P15 mice, 100 mL for adult mice) followed by 4% PFA in the same buffer (100 mL for P15 mice, 150 mL for adult mice). The brains were then removed, further fixed in 4% PFA for 1 h at 4°C, and washed in PBS (0.9% NaCl in 50 mM PBS, pH 7.4). Coronal sections, 50 μm thick, across the areas of interest were then cut in cooled PBS with a vibrating microtome (VT100S; Leica Microsystems), immersed in 0.1% sodium borohydride (Sigma-Aldrich) in PBS for 30 min at RT, and washed in this buffer before further processing.

For dual VGLUT3/SERT double-immunolabeling, five VGLUT3 WT mice of immature (P15) and of adult ages were used. Free-floating sections containing the striatum (around 0.14 mm anterior to bregma according to Paxinos and Franklin, 2008), lateral septum (around 0.14 mm anterior to bregma), ventral hippocampus (around −2.92 mm anterior to bregma) or dorsal hippocampus (around −1.64 mm anterior to bregma) were selected. Sections were preincubated for 1 h at RT in a blocking solution (PBS containing 5% normal goat serum and 0.5% gelatin) to reduce nonspecific binding. They were then incubated for 48 h in a 1:500 dilution of both primary antibodies: polyclonal goat anti-SERT (C-20, Santa Cruz Biotechnology) and polyclonal rabbit anti-VGLUT3 (Synaptic Systems). After rinses in PBS (3 × 10 min), they were placed overnight at RT in a 1:50 dilution of secondary immunogold donkey anti-goat IgGs ultrasmall (Electron Microscopy Sciences) and treated with HQ Silver^TM^ Enhancement Kit (Nanoprobes) for 10–15 min to increase the size of gold particles. The immunogold-labeled sections were then incubated for 2 h in a 1:1000 dilution of biotinylated donkey anti-rabbit (for VGLUT3) IgGs (Jakson Immunoresearch) in blocking solution. After rinses in PBS (3 × 10 min), sections were incubated for 1 h in a 1:1000 dilution of HRP-conjugated streptavidin (Jackson Immunoresearch), washed in PBS and incubated for 2–5 min in Tris-buffered saline (TBS) containing 3,3′-diaminobenzidine tetrahydrochloride (0.05% DAB) and hydrogen peroxide (0.02%). Immunocytochemical controls included processing without either one or both primary antibodies. Only the expected single labeling was observed after omitting one of the primary antibody, and no labeling was detected in the absence of both antibodies.

For single SERT immunolabeling, P15 and P60–70 VGLUT3 WT and KO mice were used (five animals per each age and genotype). Free-floating sections containing the striatum (around 0.14 mm anterior to bregma) were processed for SERT immunohistochemistry with the same pre-embedding immunogold technique described above.

All sections were further processed for electron microscopy as extensively reported in previous publications (Bérubé-Carrière et al., 2009, 2012). Briefly, sections were postfixed in 1% osmium tetroxide for 30 min, dehydrated in ascending concentrations of ethanol and flat embedded in Durcupan (Sigma-Aldrich) between two sheets of Aclar plastic (EMS). After 48 h of polymerization at 60°C, the sheets were removed and specimens from the areas of interest were excised and glued at the tip of resin blocks. Ultrathin (70–80 nm) sections were then cut from the surface of the blocks using a Reichart Ultracut S ultramicrotome (Leica), collected on bare square-mesh copper grids and stained with Reynold’s lead citrate.

#### Quantitative Analysis of Electron Microscopic Data

Five thin sections from each subregion were analyzed from each animal. Only sections at the tissue-surface interface (less than 10 μm away from the tissue-resin border) were selected for analysis to avoid any differences in labeling due to differing penetrance of reagents. The thin sections from each group were examined at a working magnification of 13,500× under a CM100 electron microscope (Philips) equipped with an AMT camera system (Advanced Microscopy Techniques). For the analysis of gold particle distributions, an axon terminal profile was considered to be positively labeled for SERT when it contained three or more silver-intensified gold particles delineating the axon terminal membrane. Smaller immunolabeled profiles were assumed to represent intervaricose segments of unmyelinated axons (Bérubé-Carrière et al., 2009). It should be pointed out that peroxidase reaction product (labeling VGLUT3 in our study) is typically not particulate but diffuse in nature, and thus not easily confused with silver-enhanced immunogold products. The acquired images were analyzed using the public domain ImageJ software from NIH. In dual labeling experiments, the following parameters were quantified for each brain subregion: (1) number of gold-silver labeled varicosities (SERT positive); (2) number of DAB-immunoreactive varicosities (VGLUT3 positive); and (3) number of dually SERT/VGLUT3 labeled varicosities. In simple labeling experiments evaluating SERT expression in the striatum, long (L), short (s) axes and mean diameter (L+ s/2) were measured for each immunogold-labeled varicosity. The percentage of mitochondria was calculated as the total number of mitochondria in all terminals divided by the examined surface in total.

#### Statistics

Data are represented throughout as mean ± SEM. Statistically significant differences were analyzed using Student’s *t* test, one-way ANOVA or two-way ANOVA with *post hoc* Tukey tests, as appropriate. A Whelch correction was applied when appropriate. For immunoelectron microscopy experiments a Kruskal-Wallis test was used when the data were found to not be normally distributed. *P*-values below 0.05 were considered statistically significant.

## Results

### VGLUT3 is Found at SERT-Negative Release Sites in Cultured 5-HT Neurons

**Figure 1:**
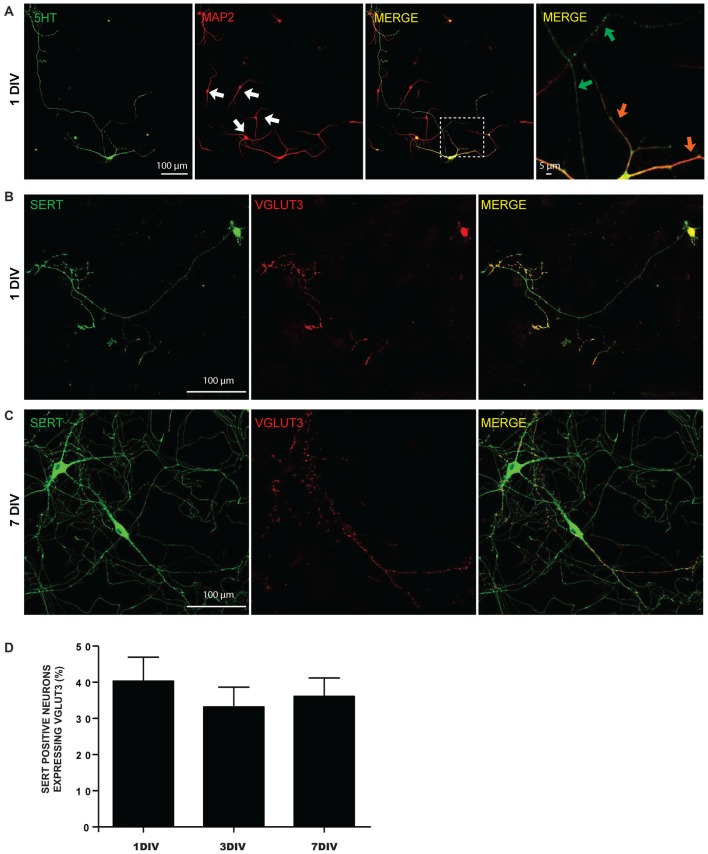
**Expression of 5-HT reuptake transporter (SERT) and vesicular glutamate transporter (VGLUT3) in cultured serotonin (5-HT) neurons. (A)** Cultured 5-HT neurons display a highly developed axonal arborization, even after 1 day *in vitro* (DIV). Photomicrographs taken with a 20× magnification illustrating 5-HT and microtubule associated protein 2 (MAP2) immunoreactivity. The white arows identify non-5-HT neurons. These illustrations show that axonal and dendritic processes of 5-HT neurons are uniformly 5-HT immunoreactive. The orange arrows in the zoomed area (identified by the white box) show 5-HT neuron dendrites, while the green arrows identify axonal domains of the same neuron. **(B)** Photomicrographs taken with a 20× magnification illustrating SERT positive neurons expressing VGLUT3 at 1 DIV. **(C)** Photomicrographs taken with a 20× magnification illustrating SERT positive neurons expressing VGLUT3 at 7 DIV. **(D)** Bar graph representing the proportion of SERT positive neurons expressing VGLUT3 protein at 1, 3 and 7 DIV. Scale bar = 100 μm (20× magnification) or 5 μm (crop).

We first examined the possibility of segregated glutamate release sites in postnatal 5-HT neurons cultured from the dorso-median part of the raphe of P0-P3 WT mice (Figure 1). Even as early as 1 DIV, raphe 5-HT neurons displayed highly branched MAP2-negative 5-HT-positive axonal processes with numerous axonal-like varicosities (Figures 1A,B). Dendrites were both MAP2- and 5-HT-positive (Figure 1A). A large majority of raphe 5-HT neurons expressed SERT, while less than half were immunopositive for VGLUT3 (Figures 1B,C). The proportion of 5-HT neurons expressing VGLUT3 remained constant at 1, 3 and 7 DIV and represented approximatively 40% of SERT-positive neurons in culture (Figure 1D).

**Figure 2:**
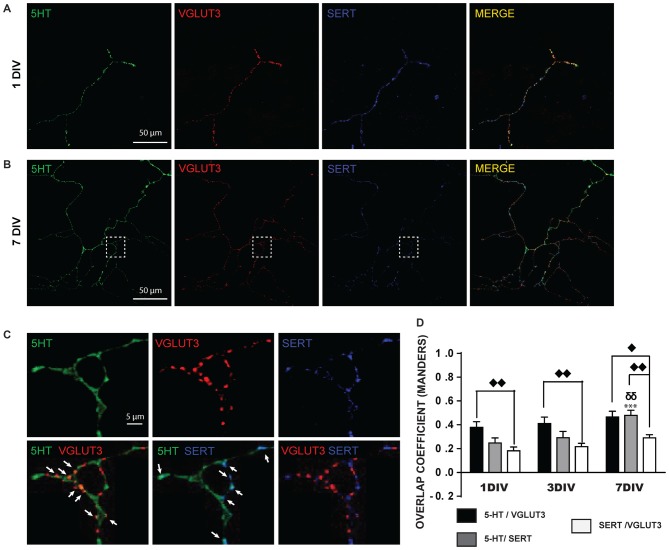
**SERT and VGLUT3 proteins are located at distinct axonal varicosities in cultured 5-HT neurons. (A)** Photomicrograph of an axonal branch from a 5-HT neuron at 1DIV, taken at 60× magnification. 5-HT (green), VGLUT3 (red) and SERT (blue) immunoreactivity is shown. **(B)** Photomicrograph for a similar 5-HT, VGLUT3, SERT triple-labeling from a 5-HT neuron at 7 DIV. **(C)** Cropped region identified by the while box in **(B)**. As can be seen, while 5-HT signal is found throughout the axonal segment, VGLUT3 and SERT are found only at a subset of axonal-like varicosities and are mostly segregated. The arrows show axon terminals coexpressing 5-HT and VGLUT3 or 5-HT and SERT. **(D)** Bar graph representing Mander’s coefficient for the three signal combinations (5-HT/VGLUT3, 5-HT/SERT and SERT/VGLUT3) in cultured 5-HT neurons after 1, 3 and 7 DIV. The results show an increase over time of the colocalization between 5-HT and SERT due to increased SERT signal over time. They also show that the colocalization of VGLUT3 with SERT is lower than with 5-HT. ^⧫^*p* < 0.05; ^δδ^ and ^⧫⧫^*p* < 0.01; ***p < 0.001. Scale bar = 50 μm (60× magnification) or 5 μm (crop).

A closer examination of the localization of 5-HT, SERT and VGLUT3 in the axonal arborization of cultured 5-HT neurons revealed that 5-HT-immunoreactivity was detectable in SERT-positive or VGLUT3-positive varicosities (Figures 2A,B). However, strikingly, VGLUT3-positive and SERT-positive axonal varicosities were frequently segregated (Figure 2C) and a high proportion of VGLUT3-positive terminals were thus essentially devoid of detectable SERT immunoreactivity. A quantitative analysis of the colocalization of these signals was performed by calculating the MOC (Figure 2D), as well as the associated M1 and M2 coefficients (Supplementary Figures S1A,B). The MOC displayed a gradual increase across the three ages examined (DIV 1, 3 and 7; Figure 2D), as reflected by a significant time effect in the two-way ANOVA (*F*_(2,99)_ = 11.99 and *p* = 0.0002). There was also a significant difference between the three signals (5-HT/VGLUT3, 5-HT/SERT and SERT/VGLUT3; *F*_(2,99)_ = 19.69 and *p* < 0.0001), but no interaction (*F*_(4,99)_ = 2.73 with *p* = 0.39). A Tukey multiple comparison *post hoc* test showed that the 5-HT/SERT MOC increased significantly at 7 DIV (MOC = 0.48) compared to 1 DIV (MOC = 0.25; *p* < 0.001) and to 3 DIV (MOC = 0.29; *p* < 0.01), probably due to an age-dependent increase in the levels of expression of SERT. We also found that VGLUT3 displayed a higher MOC with 5-HT than with SERT at 1 DIV (*p* < 0.01), 3 DIV (*p* < 0.01) and 7 DIV (*p* < 0.05) and that SERT has a stronger MOC with 5-HT than with VGLUT3 at 7 DIV (*p* < 0.01). The overlap coefficient M1 showed a time effect (*F*_(2,99)_ = 6.35 and *p* = 0.002) but no signal effect (*F*_(2,99)_ = 2.93 and *p* < 0.06) and no interaction (*F*_(4,99)_ = 0.83 with *p* = 0.51; Supplementary Figure S1A). A Tukey *post hoc* test showed that 5-HT contributed more to colocalization with SERT at 7 DIV (M1 = 0.64) compared to 3 DIV (M1 = 0.41; *p* < 0.05) and to 1 DIV (M1 = 0.39; *p* < 0.05). Finally, overlap coefficient M2 showed a time effect (*F*_(2,99)_ = 3.6 and *p* = 0.03) and a signal effect (*F*_(2,99)_ = 8.82 and *p* = 0.0003) but no interaction (*F*_(4,99)_ = 1.00 with *p* = 0.41; Supplementary Figure S1B). A Tukey *post hoc* test showed that SERT contributed more to colocalization with 5-HT at 7 DIV (M2 = 0.68) compared to 1 DIV (M2 = 0.46; *p* < 0.01). In addition, SERT contributed more to colocalization with 5-HT (M2 = 0.68) than VGLUT3 with SERT (M2 = 0.43) at 7 DIV (*p* < 0.01).

### VGLUT3 is Found at SERT-Negative Release Sites in the Axonal Varicosities of 5-HT Neurons in the Intact Brain

**Figure 3:**
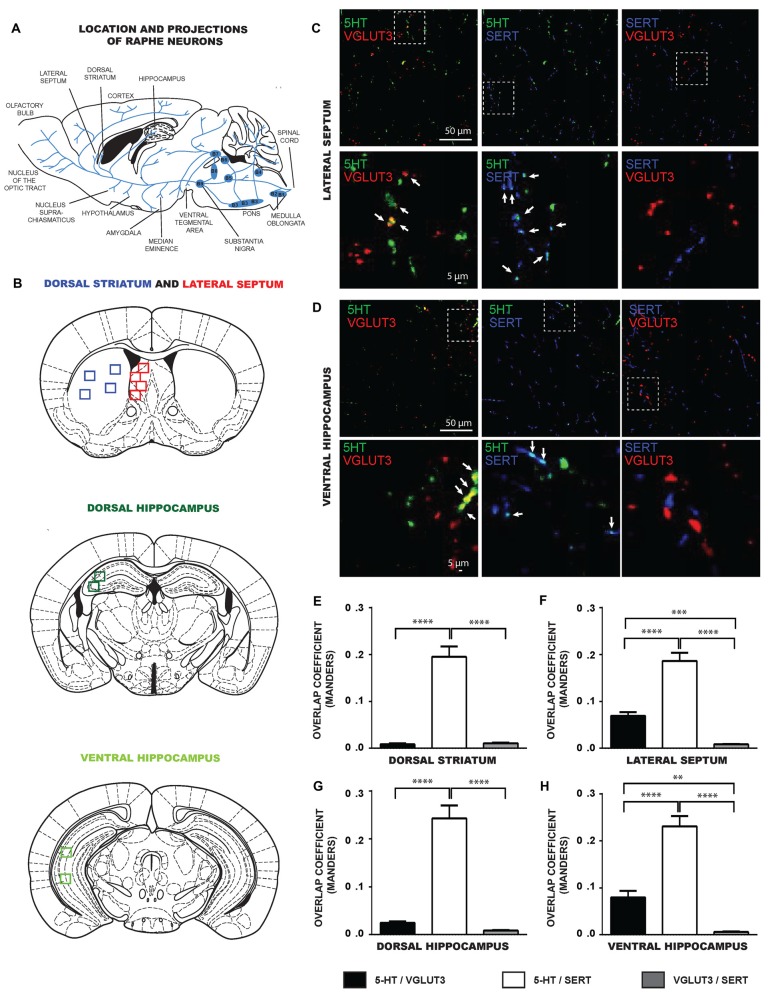
**SERT and VGLUT3 are located at distinct axonal varicosities of 5-HT neurons in intact brain tissue. (A)** Schematic illustration representing the location of different raphe nuclei and their projections. **(B)** Schematic illustrations of brain serotoninergic projection areas analyzed in the present study: lateral septum, dorsal striatum, ventral and dorsal hippocampus. **(C)** Photomicrographs taken at 60× magnification showing 5-HT axonal varicosities immunolabeled for 5-HT, SERT and VGLUT3 in the lateral septum. **(D)** Photomicrographs taken at 60× magnification showing 5-HT axonal varicosities immunolabeled for 5-HT, SERT and VGLUT3 in the ventral hippocampus. Axonal varicosities expressing both 5-HT and VGLUT3 or 5-HT and SERT are designated by arrows. **(E–H)** Bar graph representing Mander’s colocalization coefficient calculated from images captured from the dorsal striatum, lateral septum, dorsal and ventral hippocampus of P60 wild type (WT) mice. The data show that the coefficient was lowest for the colocalization of VGLUT3 and SERT, compatible with the observation that the two proteins are localized in different axonal varicosities. **p < 0.01; ***p < 0.001 and ****p < 0.0001. Scale bar = 50 μm (60× magnification) or 5 μm (crop).

We next evaluated if the limited colocalization of VGLUT3 and SERT was also found in some of the specific projection areas of 5-HT neurons in the intact tissue (Figures 3A,B). We examined brain sections from the dorsal striatum, lateral septum, dorsal and ventral hippocampus and dorsal raphe, triple-labeled for 5-HT, VGLUT3 and SERT. Similarly to *in vitro* results, confocal images revealed that 5-HT and SERT were highly colocalized in axonal varicosities in the lateral septum (Figure 3C), dorsal striatum (not shown), ventral (Figure 3D) and dorsal (not shown) hippocampus. Similarly, 5-HT and VGLUT3 were also colocalized in the lateral septum (Figure 3C) and ventral hippocampus (Figure 3D) but less so in the dorsal parts of hippocampus and striatum. However, SERT and VGLUT3 proteins were mostly found in distinct axonal varicosities in all of these projection areas.

To confirm these observations quantitatively, we analyzed the MOC of 5-HT/SERT, 5-HT/VGLUT3 and SERT/VGLUT3 in images captured from these brain regions (Figures 3E–H). Globally, the data show that the colocalization between SERT and VGLUT3 was systematically lower compared to the colocalization of either 5-HT/VGLUT3 or 5-HT/SERT. One-way ANOVA analysis identified significant differences in the MOC calculated from images obtained from the dorsal striatum (*F*_(2,96)_ = 69.34, *p* < 0.0001), lateral septum (*F*_(2,90)_ = 68.27, *p* < 0.0001), dorsal hippocampus (*F*_(2,102)_ = 69.80, *p* < 0.0001) and ventral hippocampus (*F*_(2,87)_ = 67.36, *p* < 0.0001). For the dorsal striatum (Figure 3E), a Tukey *post hoc* test showed that the 5-HT/SERT MOC was larger than the SERT/VGLUT3 MOC (*p* < 0.0001) and the 5-HT/VGLUT3 MOC (*p* < 0.0001). For the lateral septum (Figure 3F), a Tukey *post hoc* test showed that the 5-HT/SERT MOC was larger than the SERT/VGLUT3 MOC (*p* < 0.0001) or the 5-HT/VGLUT3 MOC (*p* < 0.0001). In addition, the 5-HT/VGLUT3 MOC was higher than the SERT/VGLUT3 MOC (*p* < 0.001). For the dorsal hippocampus (Figure 3G), our data revealed that the 5-HT/SERT MOC was larger than the SERT/VGLUT3 MOC (*p* < 0.0001) or the 5-HT/VGLUT3 MOC (*p* < 0.0001). For the ventral hippocampus (Figure 3H), we observed that the 5-HT/SERT MOC was larger than the SERT/VGLUT3 MOC (*p* < 0.0001) or the 5-HT/VGLUT3 MOC (*p* < 0.0001). Moreover, the 5-HT/VGLUT3 MOC was higher than the SERT/VGLUT3 MOC (*p* < 0.01).

### Ultrastructural Evidence for Segregation of VGLUT3 in Serotonergic Axon Terminals

**Figure 4:**
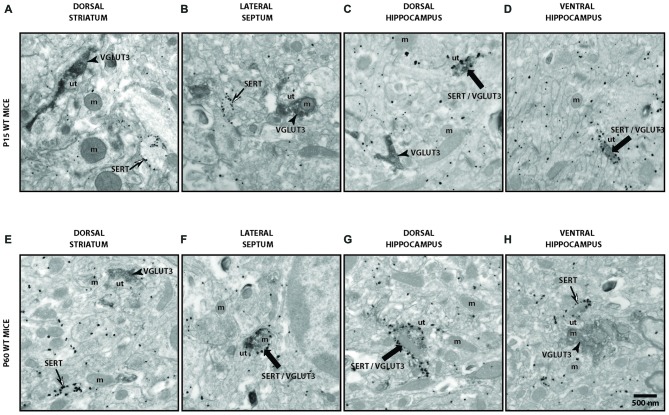
**Ultrastructural evidence for segregation of VGLUT3 and SERT in serotonergic axon terminals.** Low-magnification electron micrographs illustrating SERT- (silver-intensified immunogold particles; thin arrows), VGLUT3- (fine diaminobenzidine (DAB) precipitate; arrowheads) or SERT/VGLUT3- (silver-intensified immunogold particles associated with fine diaminobenzidine precipitate; thick black arrows) labeled axon terminals in immature (P15) **(A–D)** and adult (P60) **(E–H)** wild-type (WT) mice of striatum **(A,E)**, septum **(B,F)**, ventral hippocampus **(C,G)** and dorsal hippocampus **(D,H)**. m: mitochondria, ut: unlabeled terminal. Magnification: 13,500× , Scale bar: 500 nm.

To validate our observation of SERT/VGLUT3 segregation with a higher resolution technique, we also examined the localization of these two proteins in WT P15 and P60–70 mouse brain using double-labeling immuno-electron microscopy. Varicosities immunoreactive for SERT (immunogold labeling) and/or VGLUT3 (immunoperoxidase-DAB labeling) were examined in different brain regions at both ages (Figure 4). As previously reported, SERT-gold particles were localized mainly on the plasma membrane of labeled terminals (Descarries and Riad, 2012). Serotoninergic terminals were found in all regions explored (striatum, septum, ventral and dorsal hippocampus) from both ages (P15 and P60). As expected, terminals were filled with small, round or ovoid synaptic vesicles, often accompanied by one or more mitochondria (Figure 4). Axon terminals singly labeled for SERT or VGLUT3 were detected in all brain regions examined, namely striatum, septum, ventral and dorsal hippocampus. This was not the case for dually labeled SERT/VGLUT3 varicosities, which were only infrequently observed in the septum, ventral and dorsal hippocampus and essentially absent from the striatum (Table 1). The number of dually labeled varicosities was not statistically different between immature and adult ages. However, the number of dually labeled varicosities was significantly smaller compared to the number of singly labeled VGLUT3 terminals. As shown in Table 1, VGLUT3 was colocalized with SERT in a small proportion of all VGLUT3-labeled axon terminals in P15 and P60 mice, respectively representing 5.26% and 10.8% in the septum, 21.8% and 13.4% in the ventral hippocampus, and finally 7.6% and 7.6% in the dorsal hippocampus. Dually labeled varicosities were completely absent in the striatum of adult mice and were exceedingly rare in immature ones.

**Table 1:** **Density of SERT, VGLUT3 and SERT/VGLUT3 axon terminals in immature and mature VGLUT3 WT mice**.

	Dorsal striatum		Lateral septum		Ventral Hipp		Dorsal Hipp	
	P15	P60	P15	P60	P15	P60	P15	P60
Surface (μm^2^)	9612	6855.6	9184.8	10173.4	9754.4	7749.6	8828.8	8965.8
SERT	20.8 ± 2.1	18.6 ± 3.7	15 ± 1.7	26.4 ± 4.5	20.4 ± 3.7	26.7 ± 4.6	15 ± 3.6	22.2 ± 4.6
VGLUT3	77 ± 11.4	42.8 ± 5.2	50.4 ± 9.3	32.8 ± 6.2	24.4 ± 1.4	24 ± 7.08	38.8 ± 6.7	38.8 ± 8.2
SERT/VGLUT3	0.2 ± 0.2**	0^⧫^	2.8 ± 1.2**	4 ± 1.7^⧫^	6.8 ± 3.4**	3.7 ± 1.4^⧫^	3.2 ± 1.4**	3.2 ± 1.2^⧫^

Mean (± SEM) number of immunoreactive axon terminals per surface of thin sections examined in different brain regions after dual immunolabeling in WT mice at immature (P15) or adult (P60–70) age (n = 5 mice per age). Statistical differences were calculated on the ratio of labeled terminals per examined surface. **p < 0.01 compared to VGLUT3 in P15 mice; ^⧫^p < 0.05 compared to VGLUT3 in P60 mice.

### The Glutamate Co-phenotype of 5-HT Neurons Regulates the Vulnerability of 5-HT Neurons *In Vitro*

We next compared 5-HT neurons cultured from VGLUT3 KO mice and WT littermates in order to test the hypothesis that the glutamate co-phenotype of 5-HT neurons regulates their basal vulnerability. A fixed number of neurons were cultured for 1 or 7 DIV, and then fixed and immunostained against 5-HT (data not shown). The number of 5-HT-positive neurons after 1 and 7 DIV was compared to quantify basal survival rate. The survival rate of 5-HT neurons was determined as the number of surviving 5-HT neurons after 7 DIV compared to the number of surviving 5-HT neurons after 1 DIV. We found that the survival rate of 5-HT neurons prepared from VGLUT3 KO mice was lower than that of 5-HT neurons from WT littermates (36.59 ± 3.55% for VGLUT3 KO cultures compared to 51.82 ± 3.09% for WT cultures, *t*-test, *p* = 0.002).

### No Change in the Number of 5-HT Neurons in the Brain of VGLUT3 KO Mice

**Figure 5:**
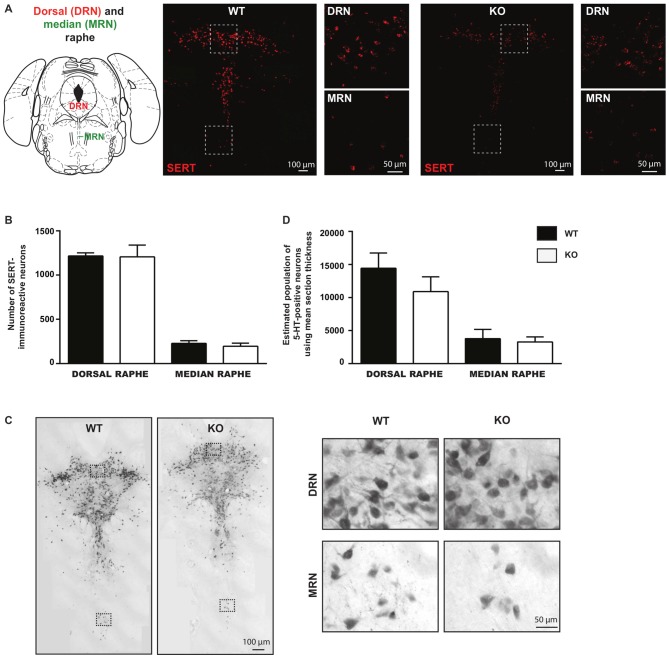
**The number of serotonin neurons is unchanged within the dorsal and median regions of the raphe nucleus in VGLUT3 KO mice. (A)** Photomicrographs taken at 20× and 60× magnifications, showing SERT mRNA in both dorsal and median raphe using fluorescent *in situ* hybridization for SERT in VGLUT3 WT and KO mice. **(B)** Bar graphs illustrating the number of SERT- mRNA-containing neurons in the dorsal and median raphe of VGLUT3 WT and KO mice. **(C)** Photomicrographs taken at 20× (left panels) and 60× (right panels) magnifications, showing 5-HT-immunoreactive neurons in both the dorsal and median raphe, revealed by DAB staining, in VGLUT3 WT and KO mice. **(D)** Bar graphs illustrating the number of 5-HT neurons in the dorsal and median raphe of VGLUT3 WT and KO mice following unbiased stereological counting. Deletion of the VGLUT3 gene did not affect the number of 5-HT neurons in the dorsal and median sub-regions of the raphe.

The observed decrease in the basal survival of 5-HT neurons *in vitro* suggests that loss of VGLUT3 leads to increased vulnerability of these neurons. We next examined whether such an increase in vulnerability was sufficient to lead to a reduced number of 5-HT neurons in the intact brain. We counted SERT-positive neurons in brain sections obtained from the DRN and MRN of WT and VGLUT3 KO mice using fluorescent *in situ* hybridization for SERT mRNA (Figure 5A). Compared to WT littermate mice, the number of SERT-positive neurons in the DRN of VGLUT3 KO mice was found to be unchanged (*t*-test; 1215 ± 35.37 (*n* = 5) for WT vs. 1206 ± 133.36 (*n* = 4) for KO mice; *p* = 0.95; Figure 5B). In the MRN, the number of SERT-positive neurons was also unchanged (*t*-test; 227.6 ± 31.18 (*n* = 5) for WT mice vs. 195.0 ± 36.19 (*n* = 4) for KO mice; *p* = 0.51; Figure 5B).

Unbiased stereological counting of the total population of 5-HT-immunoreactive neurons confirmed that deletion of the VGLUT3 gene did not result in a reduced number of 5-HT neurons in either the DRN or the MRN (Figure 5C). In DRN, the estimated number of 5-HT-positive neurons using mean section thickness was 14445 ± 2314 (*n* = 7) in WT mice vs. 10901 ± 2242 in VGLUT3 KO mice (*n* = 5; *p* = 0.31; Figure 5D). In the MRN, the estimated number of 5-HT-positive neurons using mean section thickness was 3789 ± 526.3 (*n* = 7) in WT mice vs. 3289 ± 339.6 in VGLUT3 KO mice (*n* = 5; *p* = 0.49; Figure 5D).

### Reduced Density of 5-HT-Positive Varicosities in VGLUT3 KO Brain Tissue

**Figure 6:**
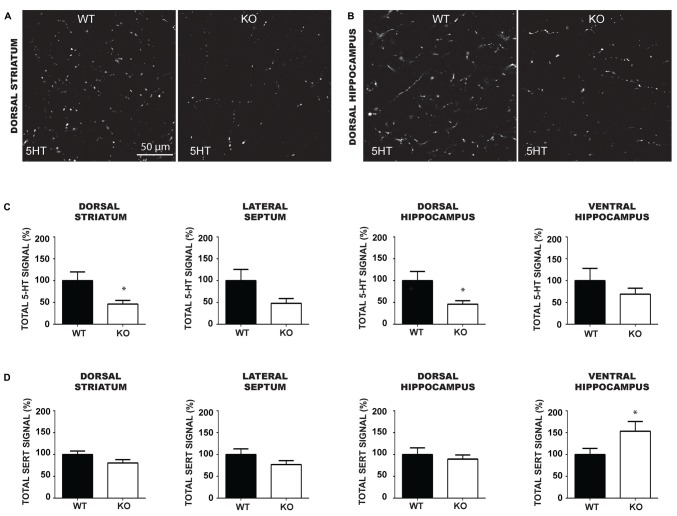
**The density of 5-HT immunoreactive terminals is reduced in the forebrain and caudal hippocampus of VGLUT3 KO mice. (A,B)** Photomicrographs taken at 60× magnification, showing 5-HT immunoreactivity in the dorsal striatum **(A)** and dorsal hippocampus **(B)** of P60 VGLUT3 WT and KO mice. **(C,D)** Bar graphs illustrating the density of 5-HT **(C)** and SERT **(D)** immunoreactivity (mean gray value × signal area) within the dorsal striatum, lateral septum, dorsal and ventral hippocampus of P60 VGLUT3 WT and KO mice. Deletion of the VGLUT3 gene reduced the total density of 5-HT-positive varicosities in the dorsal striatum and dorsal hippocampus. Finally, the total density of SERT immunoreactivity was unchanged in the forebrain and dorsal hippocampus, but increased in the ventral hippocampus of VGLUT3 KO mice. Scale bar = 50 μm (60× magnification). *p < 0.05.

Although the number of 5-HT neurons was unchanged in VGLUT3 KO mice, loss of VGLUT3 could have an impact on the axonal arborization of 5-HT neurons, similarly to what has previously shown in the absence of VGLUT2 in DA neurons (Fortin et al., 2012). To examine this possibility, we performed a 5-HT/SERT double-immunolabeling experiment to examine the axonal processes of 5-HT neurons in WT and VGLUT3 KO mice in the dorsal striatum (Figure 6A), lateral septum, dorsal hippocampus (Figure 6B) and ventral hippocampus. We quantified total 5-HT (Figure 6C) and SERT (Figure 6D) signal density by multiplying the mean signal intensity by the signal area. Results were expressed relative to the signal density of VGLUT3 WT mice. The density of 5-HT-immunoreactive varicosities was significantly reduced in the dorsal striatum (*t*-test, 100% ± 19.90 vs. 46.25% ± 8.52; *p* = 0.017) and dorsal hippocampus (*t*-test, 100% ± 20.87 vs. 46.09 ± 7.96; *p* = 0.02) of VGLUT3 KO mice compared to WT littermates, with a similar tendency in the lateral septum (*t*-test, 100% ± 25.75 vs. 48.01 ± 11.01; *p* = 0.07) and no change in the ventral hippocampus. The total density of SERT-immunoreactivity was not significantly changed in the dorsal striatum, lateral septum and dorsal hippocampus, but was increased in the ventral hippocampus (*t*-test, 100% ± 13.97 vs. 153.4 ± 22.23; *p* = 0.047) of VGLUT3 KO mice compared to WT littermates.

### The Ultrastructural Features of SERT Immunoreactive Axon Terminals are not Altered in the Striatum of Immature and Mature VGLUT3 KO Mice

**Figure 7:**
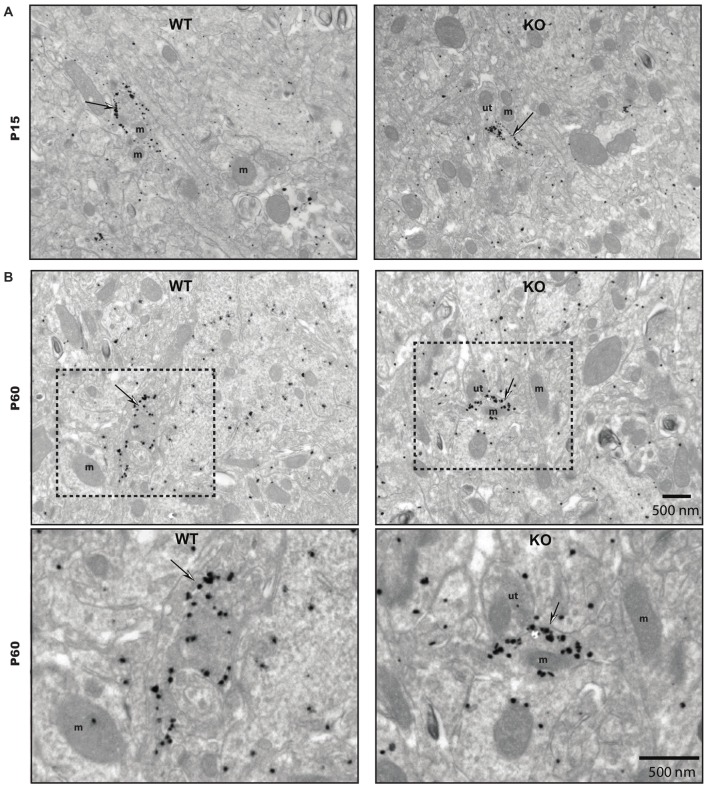
**The ultrastructural features of SERT immunoreactive axon terminals are unchanged in the striatum of immature and mature VGLUT3 KO mice.** SERT immunoreactive axon terminals in the striatum of immature (P15) **(A)** and adult (P60) **(B)**, wild-type (WT) and VGLUT3 knock-out (KO) mice, after immunogold labeling for SERT. Terminals singly labeled for SERT (thin arrows) were observed for each age and genotype and found to be unchanged in size or in the frequency at which they contain mitochondria. m, mitochondria; ut, unlabeled terminal. Magnification: 13,500×, Scale bar: 500 nm.

To gain insight into the roles of VGLUT3 and glutamate release in the structure of serotonergic axon terminals, we next used immuno-electron microscopy to examine whether the ultrastructural features of serotonergic terminals were modified in the absence of VGLUT3. We focused our attention on the striatum, the region showing the most extensive segregation of VGLUT3 and SERT. Striatal axon terminals labeled for SERT exhibited similar size and shapes for both genotypes (Figure 7). As mentioned previously, they typically contained synaptic vesicles, often accompanied by mitochondria (Figure 7). SERT-labeled terminals typically did not display a morphologically defined area of synaptic membrane specialization, compatible with previous work highlighting the mostly non-synaptic nature of these release sites (Soghomonian et al., 1989). The ultrastructural features of SERT singly labeled varicosities in the striatum of immature (Figure 7A) and mature (Figure 7B) mice are summarized in Table 2. Overall, the diameter of SERT-labeled terminal profiles ranged from 0.47 ± 0.01 μm to 0.57 ± 0.03 μm, corresponding to an area of 0.14 ± 0.002 μm^2^ to 0.2 ± 0.02 μm^2^. The proportion of labeled terminals containing one or more mitochondria ranged from 19.1 ± 2.1% to 29.51 ± 2.3%. These parameters were not statistically different between genotypes at the same age or across ages. Taken together, those data indicate the absence of VGLUT3 was not associated with a change in the morphological features of serotonergic axon terminals in the striatum of mice.

**Table 2:** **The structural features of SERT immunoreactive axon terminals in the striatum of immature (P15) and mature (P60–70), VGLUT3 KO mice are unaltered**.

			Dimensions	
	*n*	No.	Mean diameter (μm)	Area (μm^2^)	Mitochondria (%)
**P15**
WT	5	157	0.51 ± 0.04	0.17 ± 0.02	19.1 ± 2.1
KO	5	129	0.57 ± 0.03	0.2 ± 0.02	20.95 ± 3.6
**P60**
WT	5	157	0.47 ± 0.03	0.14 ± 0.01	26.85 ± 6.2
KO	5	152	0.47 ± 0.01	0.14 ± 0.002	29.51 ± 2.3

*The table provides the mean diameter and the area of SERT-positive axon terminals detected in striatal thin sections. The proportion of terminals containing mitochondria was also calculated. Data are expressed as means ± SEM. n, number of mice; No., number of axon terminals examined*.

## Discussion

Due to expression of VGLUT3, a subpopulation of raphe 5-HT neurons has the capacity to release glutamate. Studies of VGLUT3 KO mice have previously suggested that vesicular glutamate uptake in 5-HT neurons may have the capacity to facilitate vesicular uptake of 5-HT and thus regulate 5-HT-dependent behavioral states such as anxiety (Amilhon et al., 2010). However, the distribution and functional roles of glutamate release sites by 5-HT neurons remain incompletely characterized. In the present work, we further studied VGLUT3 in 5-HT neurons to address key outstanding issues. We provide evidence that VGLUT3 is found in a subset of serotonergic axon terminals that are mostly SERT-negative. Furthermore, we find that loss of VGLUT3 is associated with an increase in the basal vulnerability of 5-HT neurons *in vitro*, compatible with a critical regulatory role of glutamate release in the resilience of 5-HT neurons. We also report that deletion of VGLUT3 leads to a reduced density of 5-HT-immunoreactive axonal varicosities in projection areas of raphe 5-HT neurons, which suggests a role of glutamate in regulating either the axonal arborization of these neurons or the vesicular packaging of 5-HT.

### Heterogeneity of Axon Terminals Established By 5-HT Neurons

The ability of 5-HT neurons to release glutamate was first suggested over 20 years ago using patch-clamp recordings from isolated rat 5-HT neurons in culture (Johnson, 1994). In more recent studies, optogenetics was used to confirm that stimulation of axonal fibers originating from mouse raphe 5-HT neurons can induce fast glutamate-mediated postsynaptic currents in CA1 hippocampal pyramidal neurons (Varga et al., 2009). This glutamate release is mediated by the specific expression of VGLUT3 in 5-HT neurons, as first suggested following the initial cloning of the VGLUT3 gene and the mapping of the distribution of its mRNA in select neuronal populations including raphe 5-HT neurons (Fremeau et al., 2002; Gras et al., 2002). In an initial qualitative study of the distribution of the VGLUT3 protein in isolated rat 5-HT neurons in culture, it was reported that approximately half of the 5-HT neurons were immunopositive for VGLUT3 (Fremeau et al., 2002). Furthermore, in VGLUT3-positive 5-HT neurons, these authors reported the presence of VGLUT3 in a majority of 5-HT-positive axonal-like varicosities. A more recent study using a single-neuron tracing technique in the adult rat suggested that all 5-HT neuronal cell bodies in the dorsal raphe were VGLUT3-positive (Gagnon and Parent, 2014). They also reported that a vast majority of the axonal varicosities of 5-HT neurons in the striatum and pre-frontal cortex are co-labeled with VGLUT3. In contrast, a previous study reported that only a minority of 5-HT fibers in the adult rat ventral midbrain are VGLUT3-positive (Martín-Ibañez et al., 2006). A previous study also evaluated the distribution of VGLUT3, 5-HT and SERT in projection areas of 5-HT neurons in the mouse and reported that VGLUT3 was rarely associated with SERT positive terminals in the lateral septum, prelimbic cortex and hippocampus (Amilhon et al., 2010).

Our present findings confirm and extend previous reports hypothesizing that the axonal varicosities of 5-HT neurons are highly heterogeneous (Fremeau et al., 2002; Amilhon et al., 2010). Our *in vitro* results provide strong data showing that axonal-like varicosities established by individual 5-HT neurons are neurochemically heterogeneous, with only a subset of 5-HT-positive varicosities containing VGLUT3 or SERT. Furthermore, we found that VGLUT3 and SERT were mostly segregated in different sets of axonal-like varicosities. Our observation that in brain sections, VGLUT3 and SERT are only rarely colocalized, also argues in favor of the hypothesis of segregated release sites. However, additional experiments using techniques to label the axonal domain of single 5-HT neurons will be required to fully confirm this model *in vivo*. Our *in vitro* data demonstrating such segregation are compatible with earlier work first suggesting such heterogeneity (Amilhon et al., 2010). Our work showing that only approximately half of 5-HT neurons express VGLUT3 are also compatible with earlier work (Fremeau et al., 2002). The rarity of SERT/VGLUT3 dually-labeled endings, confirmed by our immuno-electron microscopy results, reinforces the hypothesis of the existence of a subset of 5-HT terminals displaying enhanced extracellular 5-HT levels after release due to weak 5-HT reuptake and enhanced vesicular packaging of 5-HT in VGLUT3-positive serotonergic terminals (Mestikawy et al., 2011). These findings however stand in relative contrast to previous work suggesting that essentially all raphe 5-HT neurons contain VGLUT3 and that almost all of their terminals in the striatum and cortex also contain the protein (Gagnon and Parent, 2014). The reason for this discrepancy remains unclear at the present time. Other than technical issues related to antibody specificity and sensitivity, one possibility is that the relative proportion of 5-HT terminals containing VGLUT3 may depend on the species used. Here, we examined the axonal distribution of 5-HT, SERT and VGLUT3 in the mouse, whereas Gagnon and Parent (2014) evaluated rat tissue. It is therefore possible that VGLUT3 is expressed more extensively and broadly in rat compared to mouse 5-HT neurons. Interestingly, in the P15 rat, VGLUT2 is found to be more frequently colocalized with dopaminergic markers such as tyrosine hydroxylase than in the mouse, also illustrating species differences in vesicular glutamate transporter localization in monoamine neurons (Bérubé-Carrière et al., 2009, 2012). Importantly, our finding of segregation of VGLUT3 and SERT in 5-HT neurons is compatible with a similar segregation of VGLUT2 and dopaminergic markers in DA neurons (Fortin et al., 2012; Zhang et al., 2015). Our results are also compatible with previous work also arguing for neurotransmitter segregation in other classes of neurons throughout the nervous system (Sámano et al., 2012). Similar segregation has for example been observed in the invertebrate nervous system (Sossin et al., 1990), in mammalian motor neurons (Nishimaru et al., 2005), in neurons of the bed nucleus of the stria terminalis projecting to the ventral tegmental area (Kudo et al., 2012), in sympathetic ganglia (Sámano et al., 2009) and in startburst amacrine cells of retina (Lee et al., 2010). Interestingly, previous work suggests that neurotransmitter segregation is not a fixed or invariable characteristic of neurons, but rather is a plastic phenomenon that can be influenced by the synaptic environment or by signals such as trophic factors (Vega et al., 2010, 2015).

The segregation of VGLUT3 and SERT that we observed here could potentially underlie differential release of glutamate and 5-HT from different terminals established by 5-HT raphe neurons. They might use glutamate as transmitter at VGLUT3 positive varicosities and serotonin at SERT positive varicosities and the release of one or the other transmitter could be differentially regulated under certain conditions. Transmitters segregation may confer to neurons the capability to specifically change the function of single endings following plastic experiences (Sámano et al., 2012).

Finally, it is important to consider that regions such as the striatum or hippocampus also contain VGLUT3-positive terminals that originate respectively from cholinergic neurons of the striatum or GABAergic interneurons of the hippocampus (Fremeau et al., 2002; Gras et al., 2002; Boulland et al., 2004). In particular, Boulland et al. (2004) used immunofluorescence and confocal microscopy to reveal partial co-expression of VGLUT3 and VGAT, the vesicular GABA transporter, in terminals close to pyramidal cells and granule cells in the rat hippocampus, with an increase of such co-expression during development. Interestingly, they also showed that during the early postnatal period in the rat, some VMAT2-positive/TH-negative terminals in the hippocampus co-express VGLUT3, suggesting that they represent 5-HT terminals. The detection of such terminals as VGLUT3-positive but SERT-negative in our experiments is likely to have contributed to the relative rarity of SERT/VGLUT3 terminals, but even considering this, it remains clear that SERT-positive terminals also containing VGLUT3 are the exception rather than the norm in the mouse brain.

Although further work will be required to test this hypothesis, it is possible that the segregated expression of VGLUT3 and SERT in 5-HT terminals may be accompanied by morphological heterogeneity of the axon terminals. For example, we hypothesize that VGLUT3-positive endings may be synaptic in structure whereas VGLUT3-negative/SERT-positive or VGLUT3-negative/SERT-negative 5-HT terminals may be asynaptic (Umbriaco et al., 1995; Parent et al., 2010; Mestikawy et al., 2011). Our present finding that essentially all SERT-positive axonal varicosities visualized by electron microscopy failed to show a synaptic specialization is compatible with this hypothesis. Interestingly, in the rat, it has been demonstrated that the 5-HT terminals expressing SERT in the cortex and ventral striatum were preferentially fusiform while the SERT negative terminals were larger and more spherical (Kosofsky and Molliver, 1987).

### Impact of VGLUT3 Gene Deletion on Serotonergic Axon Terminals

5-HT neurons regulate several neuronal and physiological functions like anxiety, mood, aggressive behavior, nociception, circadian rhythm as well as appetitive and sexual behaviors. Recently, Amilhon and collaborators (Amilhon et al., 2010) reported that deletion of the VGLUT3 gene in mouse leads to anxiety-like behaviors, a behavioral phenotype they attributed to reduced 5-HT vesicular packaging secondary to loss of VGLUT3. Our data showing reduced 5-HT immunoreactivity in the dorsal striatum and dorsal hippocampus of VGLUT3 KO mice support this hypothesis. The reduced 5-HT immunoreactivity we detected in the present study could be due to either of two possible mechanisms. First, this could be due to reduced concentrations of 5-HT in serotonergic axon terminals due to reduced vesicular packaging, as per the vesicular synergy hypothesis (Gras et al., 2008; Amilhon et al., 2010). Second, this could be due to an impaired axonal development of 5-HT neurons, perhaps similar to the impairment of axonal growth reported in DA neurons after selective deletion of the VGLUT2 gene (Fortin et al., 2012). Our *in vitro* results showing no impairment or axonal (Supplementary Figures S2A,C) or dendritic (Supplementary Figures S2B,D) growth in 5-HT neurons in the absence of VGLUT3 argue in favor of the first hypothesis.

We found that 5-HT immunoreactivity was not significantly decreased in the lateral septum and ventral hippocampus in VGLUT3 KO mice. Moreover, this was associated with an increased density of SERT immunoreactivity in the ventral hippocampus. Selective upregulation of SERT expression in the ventral, but not in the dorsal part of hippocampus of VGLUT3 KO mice might suggest that hippocampal SERT transporters vary in their adaptability to changes in 5-HT tone along the dorsoventral axis. The reason for this differential effect in the ventral hippocampus is presently unclear. The density of 5-HT innervation in this structure was lower than the other brain regions examined. This could have led to increased variability of the measurements and reduced statistical power. Alternately, up-regulation of SERT in the ventral hippocampus could represent a paradoxical compensatory response to low 5-HT levels (Hamon et al., 1981).

The lack of changes in the density or ultrastructure of SERT-positive varicosities observed here could reflect the fact that loss of VGLUT3 in 5-HT neurons did not lead to a developmental reduction of the growth capacity of serotonergic terminals, as suggested by our *in vitro* results. Rather, the reduced density of 5-HT immunoreactivity could reflect reduced vesicular packaging of 5-HT, due to loss of vesicular synergy. Further work will be required to demonstrate this, such as by tracing the axonal arborization of individual 5-HT neurons *in vivo* using virally-encoded tracers.

Our *in vitro* comparison of the basal survival of 5-HT neurons in VGLUT3 KO cultures revealed reduced survival compared to WT littermate controls. These findings, suggesting increased vulnerability, are compatible with a similar observation made for DA neurons after deletion of the VGLUT2 gene (Fortin et al., 2012). These results suggest the possibility that glutamate release may play a pro-survival role in monoamine neurons in general. However, considering our finding of a lack of decrease in the number of 5-HT neurons in the MRN and DRN of VGLUT3 KO mice, as determined by unbiased stereological counting and fluorescent *in situ* hybridization, our *in vitro* observations suggest the possibility that although basal survival may be unchanged in VGLUT3 KO mice, 5-HT neurons in these animals may be more vulnerable to cellular stress, such as in the context of Parkinson’s disease, a pathology in which a subset of 5-HT neurons are lost in addition to DA neurons. Further experiments evaluating the vulnerability of 5-HT neurons to neurotoxins such as 5,7-dihydroxytryptamine would help to further address this possibility.

In conclusion, our data provide strong support for the hypothesis that 5-HT neurons express a highly heterogeneous set of axon terminals, only a subset of which contains VGLUT3 or SERT. The almost complete segregation of SERT and VGLUT3 is compatible with the possibility that this morphological heterogeneity is associated with a functional heterogeneity such that terminals expressing VGLUT3 but not SERT may be sites of elevated 5-HT neurotransmission due to increased vesicular packaging and reduced extracellular reuptake. These findings offer a new perspective on the functional connectivity of a cell population involved in brain diseases such as anxiety disorders and depression. Further work will be required to determine whether changes in the expression and localization of VGLUT3 in 5-HT neurons are associated with disease.

## Author Contributions

ANV, OM-F, and NG: contributed to performing some of the experiments, analyzed data and contributed to writing the manuscript. GMF: contributed to performing some of the experiments, analyzed data and proof-reading the manuscript. EV: contributed to performing some of the experiments. SEM: provided experimental reagents, contributed to the planning and analysis of some of the experiments and to the writing of the manuscript. LD: contributed to the planning of some of the experiments. L-éT: contributed to the planning of the experiments and to writing the manuscript.

## Funding

This study was supported by a discovery grant from the National Sciences and Engineering Research Council (NSERC, Award No. RGPIN-2015-05230) of Canada to LD and L-éT.

## Conflict of Interest Statement

The authors declare that the research was conducted in the absence of any commercial or financial relationships that could be construed as a potential conflict of interest.

## Supplementary Material

SUPPLEMENTARY FIGURE S1 **SERT and VGLUT3 proteins are located at distinct axonal varicosities in cultured 5-HT neurons. (A)** Bar graph representing the overlap coefficient M1 for the three signal combinations (5-HT/VGLUT3, 5-HT/SERT and SERT/VGLUT3) in cultured 5-HT neurons after 1, 3 and 7 DIV. The results show an increase over time of the colocalization between 5-HT and SERT due to increased 5-HT signal over time. **(B)** Bar graph representing the overlap coefficient M2 for the three signal combinations (5-HT/VGLUT3, 5-HT/SERT and SERT/VGLUT3) in cultured 5-HT neurons after 1, 3 and 7 DIV. The results show an increase over time of the colocalization between 5-HT and SERT starting from 3 DIV due to increased SERT signal over time. They also show that the colocalization of VGLUT3 with SERT is lower than with 5-HT. *(compared to 1 DIV) and ^δ^ (compared to 3 DIV) *p* < 0.05; ^δδ^ and ^⧫⧫^*p* < 0.01.

SUPPLEMENTARY FIGURE S2 **Deletion of the VGLUT3 gene does not alter the axonal and dendritic morphology of cultured 5-HT neurons. (A)** Bar graphs illustrating the number of processes and total length of axonal processes of cultured 5-HT neurons from VGLUT3 WT and KO mice after 1 DIV. **(B)** Bar graphs illustrating the number of processes and total length of dendritic processes of cultured 5-HT neurons from VGLUT3 WT and KO mice after 1 DIV. **(C)** Bar graphs illustrating the number of processes and total length of axonal processes of cultured 5-HT neurons from VGLUT3 WT and KO mice after 7 DIV. **(D)** Bar graphs illustrating the number of processes and total length of dendritic processes of cultured 5-HT neurons from VGLUT3 WT and KO mice after 7 DIV.
