# Supplementary figures and images for: Axonal Segregation and Role of the Vesicular Glutamate Transporter VGLUT3 in Serotonin Neurons
Source: Front Neuroanat. 2016 Apr 12;10:39. doi: 10.3389/fnana.2016.00039 (PMC4828685; doi:10.3389/fnana.2016.00039)

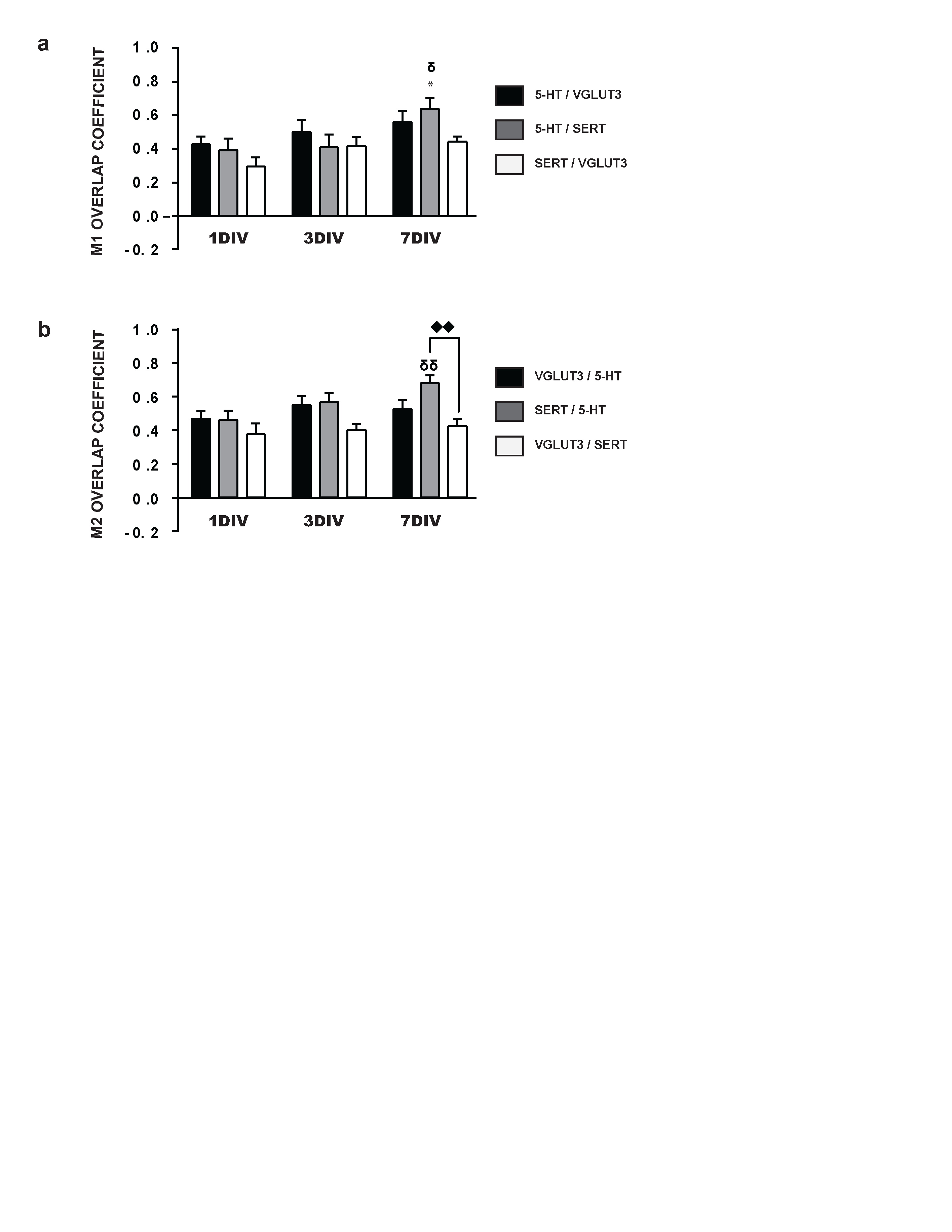

Supplement: SUPPLEMENTARY FIGURE S1 — SERT and VGLUT3 proteins are located at distinct axonal varicosities in cultured 5-HT neurons. (A) Bar graph representing the overlap coefficient M1 for the three signal combinations (5-HT/VGLUT3, 5-HT/SERT and SERT/VGLUT3) in cultured 5-HT neurons after 1, 3 and 7 DIV. The results show an increase over time of the colocalization between 5-HT and SERT due to increased 5-HT signal over time. (B) Bar graph representing the overlap coefficient M2 for the three signal combinations (5-HT/VGLUT3, 5-HT/SERT and SERT/VGLUT3) in cultured 5-HT neurons after 1, 3 and 7 DIV. The results show an increase over time of the colocalization between 5-HT and SERT starting from 3 DIV due to increased SERT signal over time. They also show that the colocalization of VGLUT3 with SERT is lower than with 5-HT. *(compared to 1 DIV) and δ (compared to 3 DIV) p < 0.05; δδ and ⧫⧫p < 0.01. [file Image_1.tif]
